# LncPep: A Resource of Translational Evidences for lncRNAs

**DOI:** 10.3389/fcell.2022.795084

**Published:** 2022-01-24

**Authors:** Teng Liu, Jingni Wu, Yangjun Wu, Wei Hu, Zhixiao Fang, Zishan Wang, Chunjie Jiang, Shengli Li

**Affiliations:** ^1^ Precision Research Center for Refractory Diseases, Institute for Clinical Research, Shanghai General Hospital, Shanghai Jiao Tong University School of Medicine, Shanghai, China; ^2^ Department of Gynecological Oncology, Fudan University Shanghai Cancer Center, Shanghai, China; ^3^ Department of Genetics and Genomic Sciences, Center for Transformative Disease Modeling, Tisch Cancer Institute, Icahn Institute for Data Science and Genomic Technology, Icahn School of Medicine at Mount Sinai, New York, NY, United States; ^4^ Institute for Diabetes Obesity, and Metabolism, Perelman School of Medicine at the University of Pennsylvania, Philadelphia, PA, United States

**Keywords:** lncRNA, peptide, translation, cancer, m6A, ribo-seq

## Abstract

Long noncoding RNAs (lncRNAs) are a type of transcript that is >200 nucleotides long with no protein-coding capacity. Accumulating studies have suggested that lncRNAs contain open reading frames (ORFs) that encode peptides. Although several noncoding RNA-encoded peptide-related databases have been developed, most of them display only a small number of experimentally validated peptides, and resources focused on lncRNA-encoded peptides are still lacking. We used six types of evidence, coding potential assessment tool (CPAT), coding potential calculator v2.0 (CPC2), N6-methyladenosine modification of RNA sites (m6A), Pfam, ribosome profiling (Ribo-seq), and translation initiation sites (TISs), to evaluate the coding potential of 883,804 lncRNAs across 39 species. We constructed a comprehensive database of lncRNA-encoded peptides, LncPep (http://www.shenglilabs.com/LncPep/). LncPep provides three major functional modules: 1) user-friendly searching/browsing interface, 2) prediction and BLAST modules for exploring novel lncRNAs and peptides, and 3) annotations for lncRNAs, peptides and supporting evidence. Taken together, LncPep is a user-friendly and convenient platform for discovering and investigating peptides encoded by lncRNAs.

## Introduction

Long noncoding RNAs (lncRNAs) are defined as RNAs longer than 200 nucleotides (nt) and have been shown to be extensively expressed and exert powerful regulatory functions (Marchese et al., 2017). Mechanistically, lncRNAs can regulate protein-protein and protein-DNA interactions by serving as scaffolds or guides, binding to proteins as decoys and modulating mRNA expression as microRNA (miRNA) sponges. Evidence accumulated over the past decade demonstrates that lncRNA regulation plays key roles in diverse biological and pathological contexts, such as the immune response (Chen et al., 2017), cell proliferation (Li et al., 2018), neuronal disorders (Salta and De Strooper, 2017), and tumour biology (Liu et al., 2021). LncRNAs have been regarded as “junk RNAs” and have no potential to encode functional proteins. Recently, a growing amount of evidence has demonstrated that lncRNAs are able to encode functional peptides that play vital roles in physiological processes (Anderson et al., 2015; Matsumoto et al., 2017; Anastasia et al., 2019; Niu et al., 2020; Cai et al., 2021; Zhang et al., 2021). For example, the translated peptides from lncRNA *Aw112010* are essential for the orchestration of mucosal immunity during bacterial infection and colitis (Jackson et al., 2018). Matsumoto *et al.* identified and functionally characterized a novel polypeptide encoded by the lncRNA LINC00961(Matsumoto et al., 2017). A LINC00961-encoded peptide was found to negatively regulate mTORC1 activation by interacting with lysosomal v-ATPase and stimulating amino acids, which further promoted muscle regeneration. The lncRNA HOXB-AS3 was discovered to encode a conserved 53 amino acid (aa) peptide that suppresses colon cancer growth by competitively binding to the arginine residues in the RGG motif of hnRNP A1 (Huang et al., 2017). These studies expand our understanding of lncRNAs and the coding potential of the genome. With increasing numbers of experimentally validated lncRNA-encoded peptides, a comprehensive identification and annotation of peptides translated from lncRNAs is urgently needed.

Various computational algorithms and biotechnologies have been developed to directly or indirectly capture translational evidence of RNAs. The coding potential assessment tool (CPAT) (Wang et al., 2013) and coding potential calculator v2.0 (CPC2) (Kang et al., 2017) are the most commonly used algorithms to assess RNA coding ability. Ribosome profiling (Ribo-seq) is a common method to identify translated RNAs (Ingolia et al., 2009), as well as the N6-methyladenosine modification of RNA (m6A) that promotes RNA translation initiation (Meyer et al., 2015), and the translation initiation site (TIS) detected by global translation initiation sequencing is important evidence for encoding proteins or peptides (Lee et al., 2012). Ribo-seq, m6A sites, and TIS provide indirect proof of lncRNA-encoded peptides. Although there is other indirect evidence supporting lncRNA-encoded peptides, none of these lines of evidence offers dependable predictions by themselves.

Several databases have annotated a few lncRNAs (Ma et al., 2019; Volders et al., 2019; Zhao et al., 2021), but a comprehensive database for translatable lncRNA annotation is still lacking. Some existing databases, for example Funcpep (Dragomir et al., 2020), ncEP (Liu et al., 2020), cncRNAdb (Huang et al., 2021), OpenProt (Brunet et al., 2021), and SmProt (Hao et al., 2018), include a fraction of lncRNA encoded information. However, Funcpep, ncEP, and cncRNAdb only collected experimentally validated peptides for a very limited number of lncRNAs; OpenProt predicts and annotates ORFs with MS, Ribo-seq, and conservation information, but only includes 10 species; SmProt did not provide related Ribo-seq, m6A, and TIS evidence for peptides and focused on peptides shorter than 100 amino acids. Some lncRNA encoded functional peptides are longer than 100 aa (Lun et al., 2020; Meng et al., 2020; Cai et al., 2021); for example, one 153 aa peptide encoded by LOC90024 promotes “cancerous” RNA splicing and tumorigenesis (Meng et al., 2020).

To identify the peptides encoded by lncRNAs, we built a comprehensive database, LncPep, that contains 10, 580, 228 peptides that were predicted to be translated from 883,804 lncRNAs across 39 species. Direct and indirect evidence is integrated to evaluate the peptide-encoding potential of lncRNAs. This database provides a convenient data search and browse engine, detailed information on each lncRNA and its translated peptide, and supporting evidence. Moreover, prediction and BLAST searches for novel lncRNAs and peptides are available for users. LncPep is expected to serve as an important resource to discover and investigate biologically functional peptides hidden in lncRNAs. All the information and data are freely accessible at http://www.shenglilabs.com/LncPep/.

## Results

### Data Source and Summary

**FIGURE 1:**
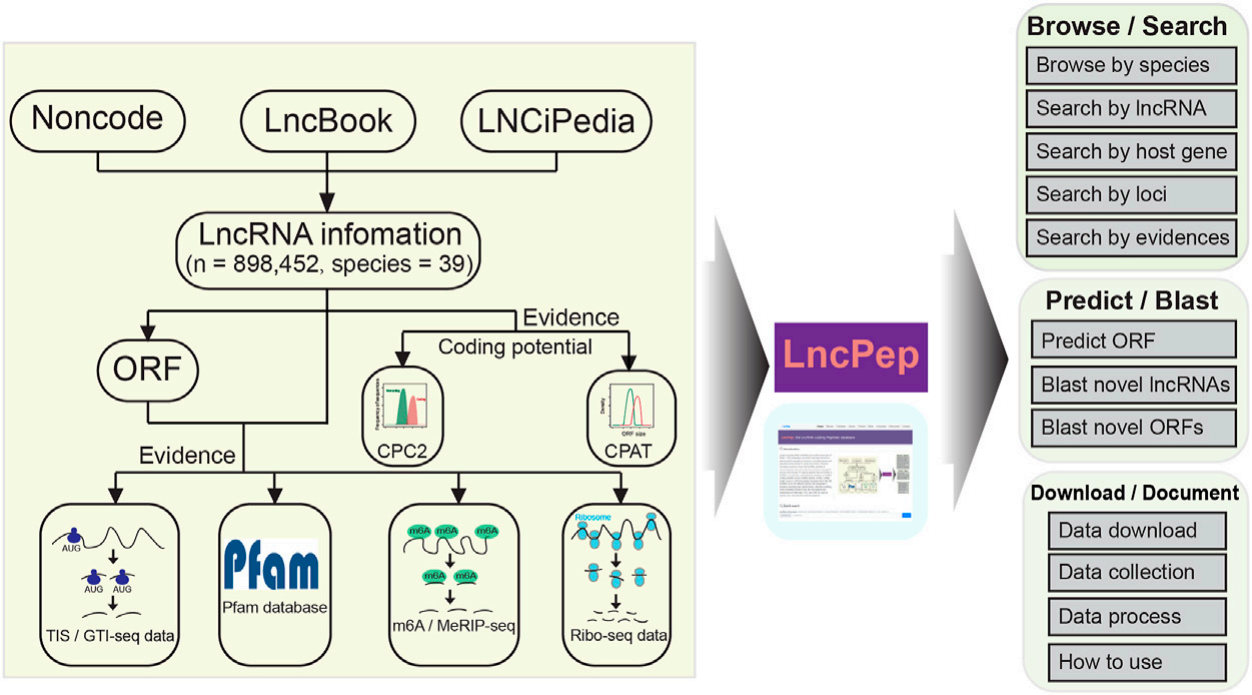
Data curation and construction pipeline of the LncPep database. LncPep collects lncRNA information from NONCODE, LncBook, and LNCipedia, including 898,452 lncRNAs across 39 different species. The peptide-translating potential of all lncRNAs was evaluated by five different tools/resources, including CPC2, CPAT, m6A, Ribo-seq, and TIS. The major functions of LncPep include Browse, Search, Predict, Blast, Download, and Document.

The current version of LncPep contains 883,804 lncRNAs across 39 species together with six different peptide-encoding lines of evidence to evaluate their translation potential (Figure 1). This evidence provides direct or indirect support for lncRNA translation. For convenience, we normalized a score for each line of evidence ranging from 0 to 1 and combined these scores for a comprehensive translation potential evaluation (see Materials and Methods).

The numbers of lncRNAs, predicted peptides, and supported lines of evidence in each species are summarized in Table 1. Detailed information on lncRNAs was retrieved from NONCODE, LncBook, and LNCipedia (Figure 1). Both ATG and non-ATG were considered start codons in all the predicted ORFs. The ORF length was set to ≥10 aa, and the longest ORF was selected when multiple ORFs overlapped in the same lncRNAs. Five different pieces of evidence to support translation are included in LncPep (Figure 1), including CPAT, CPC2, Ribo-seq, TISs, and m6A sites. Only humans and mice have five pieces of evidence, while other species have three or fewer pieces of evidence (Figure 1). The CPAT and CPC2 algorithms are the most commonly used tools for RNA coding potential evaluation, and these two tools provided the coding probability scores that were used in LncPep as evidence (Wang et al., 2013; Kang et al., 2017). Since ribosomes and TISs are necessary for RNA translation, we used Ribo-seq and validated TISs as two pieces of evidence to support lncRNA translation (Ramakrishnan, 2002; Wan and Qian, 2014; Wang et al., 2019). m6A modification was reported to promote RNA translation, and the detected m6A sites were also used as evidence (Liu et al., 2019; Meyer, 2019). “Natural” peptides are more likely to be functional, and we used the Pfam domain to assess lncRNA-translated peptides as one evidence of functionality (Mistry et al., 2021).

**TABLE 1:** Summary of lncRNAs, peptides, and evidences across 39 species.

Species	lncRNAs	Peptides	CPAT	CPC2	m6A	Pfam	Ribos	TIS
A. thaliana	3,858	21,247	3,334	3,858	2,613	214	57	240
Apple	1779	16,926	1,614	1779	N/A	261	N/A	N/A
B. napus	8,123	82,712	7,489	8,123	N/A	1740	N/A	N/A
B. rapa	6,206	67,935	5,615	6,206	N/A	1,157	N/A	N/A
Banana	1791	45,255	1,688	1791	N/A	744	N/A	N/A
*C. Elegans*	2,963	12,088	2,335	2,963	N/A	954	3,248	N/A
C. reinhardtii	771	3,876	638	771	N/A	15	N/A	N/A
Cacao	3,458	33,875	3,239	3,458	N/A	41	N/A	N/A
Cassava	5,502	233,129	5,135	5,502	N/A	4,596	N/A	N/A
Chicken	12,617	84,258	11,250	12,617	N/A	358	4,562	N/A
Chimpanzee	17,619	134,692	14,335	17,619	177	1758	N/A	N/A
Cow	21,978	97,526	17,500	21,978	N/A	201	N/A	N/A
Cucumber	2,466	19,019	2,225	2,466	N/A	112	N/A	N/A
Fruitfly	41,279	534,143	34,694	41,279	3,169	6,632	N/A	N/A
G. raimondii	1,154	5,578	948	1,154	N/A	40	N/A	N/A
Gorilla	17,886	111,120	15,451	17,886	N/A	824	N/A	N/A
Grape	3,314	173,447	3,138	3,314	N/A	4,153	N/A	N/A
Human	339,490	4,984,213	317,169	339,490	6,625	30,081	98,051	10,686
M. *truncatula*	2,177	18,751	1990	2,177	N/A	125	N/A	N/A
Maize	4,567	28,712	4,014	4,567	N/A	113	N/A	N/A
Mouse	218,223	2,571,605	122,199	218,223	5,769	11,453	24,838	5,735
O. rufipogon	7,383	104,241	6,658	7,383	N/A	1,374	N/A	N/A
O. sativa	1,118	6,702	967	1,118	N/A	33	N/A	N/A
Opossum	26,623	158,615	23,155	26,623	N/A	976	N/A	N/A
Orangutan	14,833	87,856	12,878	14,833	N/A	779	N/A	N/A
P. patens	458	3,408	421	458	N/A	38	N/A	N/A
P. trichocarpa	2,207	15,322	1978	2,207	N/A	94	N/A	N/A
Pig	29,252	261,535	26,342	29,252	N/A	390	N/A	N/A
Platypus	10,979	52,770	9,055	10,979	N/A	223	N/A	N/A
Potate	2,964	24,356	2,585	2,964	N/A	156	N/A	N/A
Quinoa	9,675	155,336	8,867	9,675	N/A	1,596	N/A	N/A
Rat	24,793	142,444	22,787	24,793	4,125	4,212	5,500	N/A
Rhesus	9,059	62,026	8,229	9,059	N/A	474	N/A	N/A
Soybean	2,209	29,999	2033	2,209	N/A	626	N/A	N/A
Tomato	3,742	89,879	3,497	3,742	N/A	1,286	N/A	N/A
Trefoil	4,969	26,120	4,223	4,969	N/A	297	N/A	N/A
Wheat	11,534	51,776	9,590	11,534	N/A	238	N/A	N/A
Yeast	50	233	37	50	76	6	N/A	N/A
Zebrafish	4,735	27,503	4,239	4,735	1,415	283	10,526	N/A

Notes: CPAT: coding-potential assessment tool, CPC2: coding potential calculator v2.0, m6A: N6-methyladenosine modification of RNA, Pfam: Protein families database, Ribos: ribosome profiling, and TIS: translation initiation site.

### LncRNA Translating Features

**FIGURE 2:**
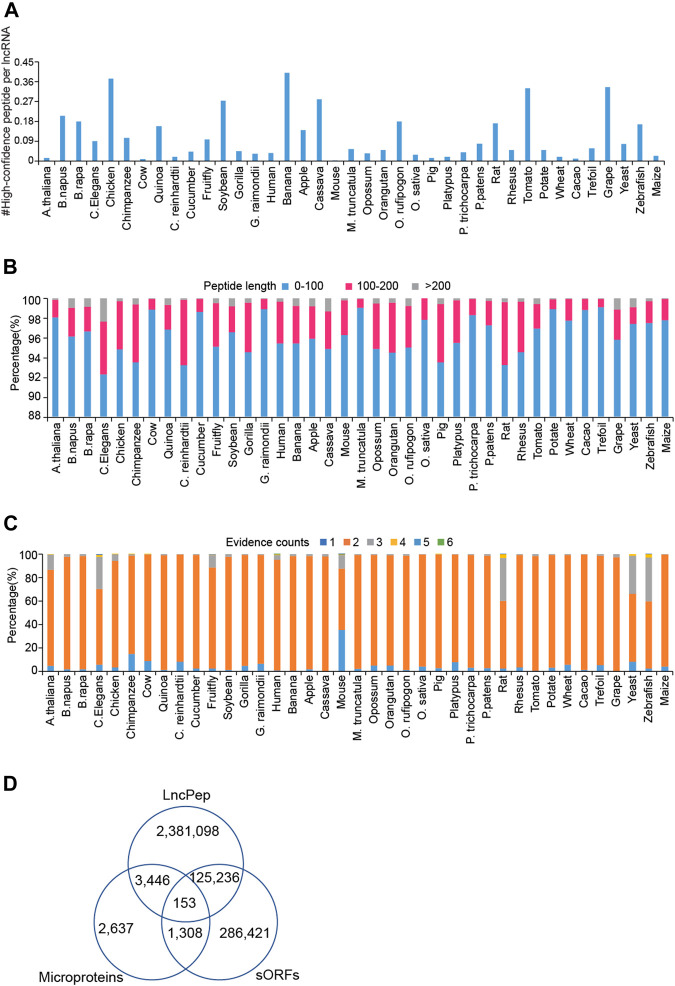
Characterization of lncRNA-encoded peptides across different species. **(A)** The distribution of high-confidence ORFs per lncRNA across 39 species. **(B)** Bar plots show the length distribution of lncRNA-encoded peptides in each species. **(C)** The distribution of supporting evidence of lncRNA-encoded peptides across different species. **(D)** The comparison of peptides among LncPep, sORFs, and Microproteins.

Peptides were predicted from extracted lncRNA sequences based on ORF searching, and translating evidence scores were calculated for predicted peptides. We defined high-confidence peptides (HCPs) as peptides with Ribo-seq evidence in human and mouse, with no less than 4 pieces of evidence in *Arabidopsis thaliana*, *Caenorhabditis elegans*, fruit fly, rat, yeast, and zebrafish, and with no less than 3 pieces of evidence in the other species. On average, less than one HCP was encoded per lncRNA in all species (Figure 2A). Although the numbers of HCPs per lncRNA in humans and mice were 0.016 and 0.01, humans and mice have a large number of lncRNAs, which makes HCPs occupy a considerable part of the human and mouse proteome. Most of the lncRNA-encoded peptides were less than 100 aa in length (Figure 2B). For evidence, the vast majority of peptides are supported by more than two pieces of evidence (Figure 2C). In humans, approximately 5% of peptides are supported by more than 2 types of evidence. We compared predicted peptides in LncPep with those in sORFs and Microproteins. Only 153 peptides were shared by all databases, and about 95% of LncPep peptides were unique in these three databases.

### Data Access and Download

**FIGURE 3:**
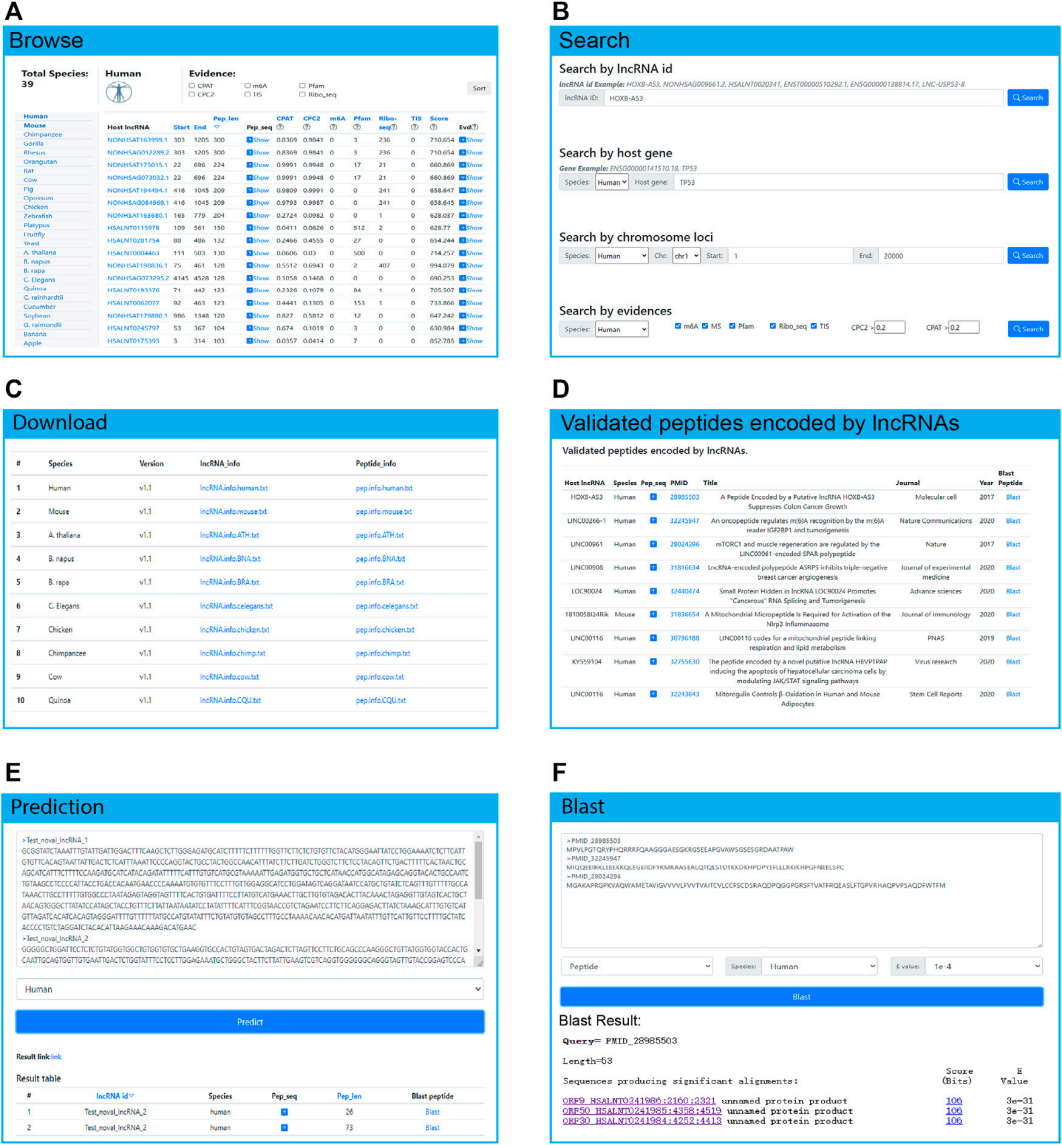
The functional modules of the LncPep database. **(A)** Users can browse the whole database by species. **(B)** The search page provides a search of lncRNA IDs, host genes, chromosome loci, and evidence. **(C)** All datasets deposited in LncPep are free to download. **(D)** The experimentally validated peptides were collected and browsed. **(E,F)** A prediction and BLAST search service was provided to explore novel lncRNAs and peptides. Species and e-values are set as parameters to choose.

LncPep provides convenient and flexible routes to mine the data. In the “Browse” module, users can select the species they are interested in, and a brief summary of the peptides will be provided, including the host lncRNA, peptide sequence and length, the evidence and the scores (Figure 3A). Users can further browse summarized details of host lncRNAs by clicking the lncRNA ID. A popup window of peptide sequences will appear by clicking the arrow in the “Pep_seq” column. Detailed evidence supporting peptides of interest will be shown after clicking the arrow in the “Evd” column. The summary table can be flexibly browsed by ranking peptide length, CPAT scores, CPC2 scores, m6A numbers, Pfam numbers, Ribo-seq numbers, TIS numbers, or integrated peptide-encoding scores. In addition, users can filter the summary table by selecting single or multiple pieces of evidence.

**FIGURE 4:**
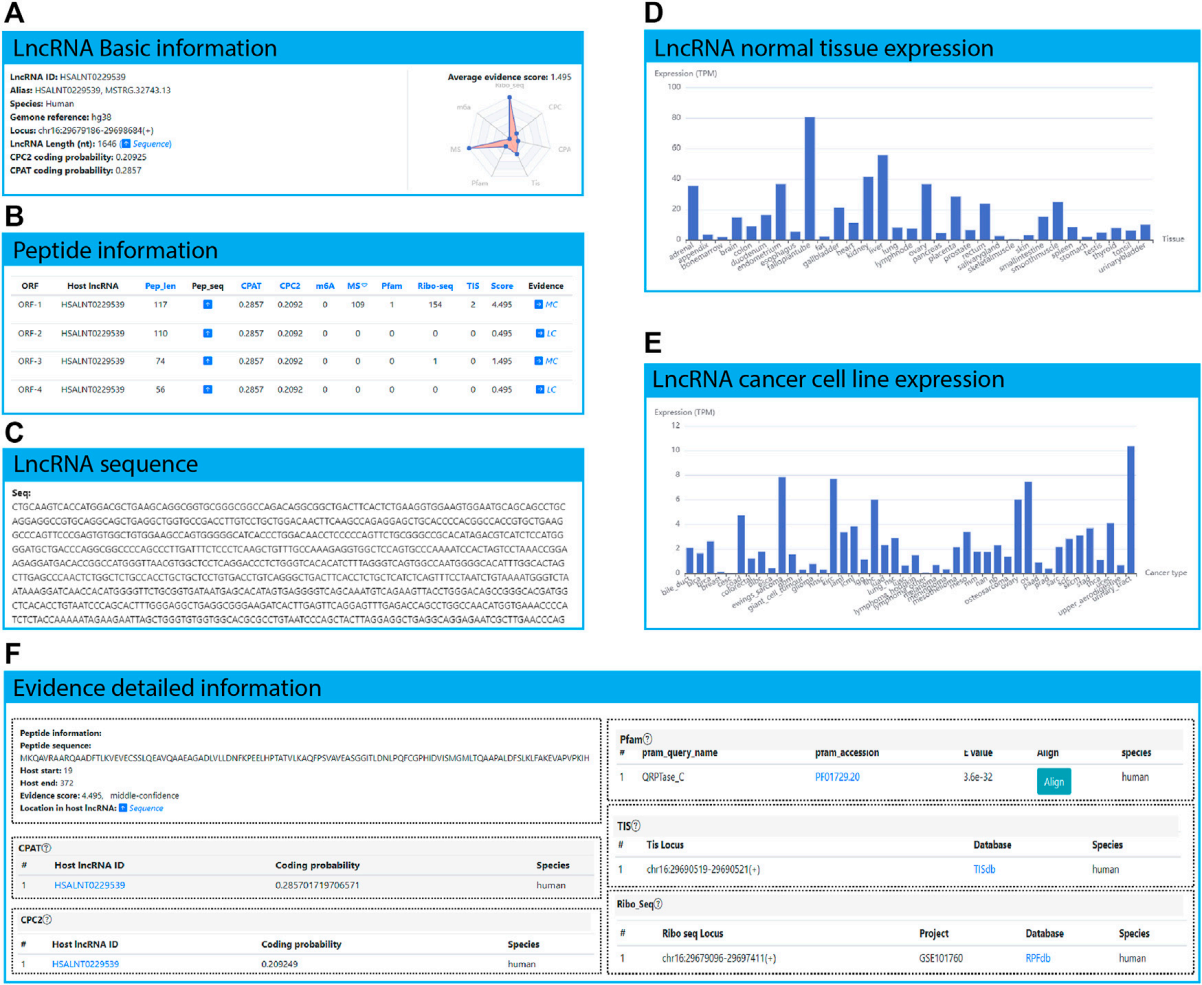
HSALNT0275775 as an example application of the LncPep database. **(A)** Basic information of lncRNA HSALNT0275775, including lncRNA ID, alias, species, genome reference, genomic locus, and length. **(B)** The summarized table of ORF and peptide information of lncRNA HSALNT0275775. **(C)** The RNA sequence and length of lncRNA HSALNT0275775 shown in the popup window by clicking the “sequence” arrow in **(A)**. **(D)** The expression levels of lncRNA HSALNT0275775 across 32 different normal human tissues. **(E)** The expression levels of lncRNA HSALNT0275775 across 44 different human cancer cell lines. **(F)** Detailed evidence of the selected peptide of lncRNA HSALNT0275775.

LncPep allows users to search the entire database by lncRNA ID, host gene, genomic location, and evidence on the search page (Figure 3B). The results table will contain peptide numbers, query names, species, lncRNA IDs, ORF genomic loci, peptide lengths, peptide sequences, ORF start sites, ORF end sites, translation scores, and supporting evidence. Search results can be ranked by peptide length, ORF start sites, and integrated peptide-encoding scores by clicking the corresponding table header names. On the lncRNA or peptide page, detailed information on the lncRNAs, peptides, and evidence is provided (Figure 4). All the data are free to download on the “Download” page (http://www.shenglilabs.com/LncPep/#!/download) (Figure 3C).

As a growing number of lncRNA-encoded peptides have been reported, we also curated experimentally validated lncRNA-encoded peptides. Through literature research and integration, we collected experimentally validated peptides from 27 articles and applied detailed information for the host lncRNAs, peptides, and articles (Figure 3D). Most of the studies were based on human lncRNAs, and another small group was based on mice. This module will continue to be updated.

### Predict and BLAST

With the development of high-throughput sequencing technology, a large number of lncRNAs and peptides have been or will be discovered. Prediction and BLAST modules will be useful for users to identify their own functional lncRNAs and peptides. Thus, we developed the “Predict” (Figure 3E) and “Blast” modules (Figure 3F) in the LncPep database, wherein users can input their own lncRNA sequences in *Fasta* format. The results table contains peptide numbers, lncRNA IDs, species, ORF numbers, ORF sequences, and options for BLAST. Users can view the ORF sequences and lengths in a popup window by clicking the arrow in the “ORF sequence” column. Users are also allowed to BLAST interested ORFs by clicking “Blast ORF” in the “Blast” column. Furthermore, users can BLAST specific lncRNA or ORF sequences based on datasets deposited in LncPep. LncRNA or ORF sequences in *Fasta* format are required for input. Before clicking the “Blast” button, users are also required to indicate whether inputting sequences are peptides or lncRNAs. The species and threshold E values are available for the user to select. Currently, up to 1,000 sequences are allowed to be uploaded and analysed at the same time, and results should be obtained within a few minutes.

### Example Application

Users can investigate potential translated peptides of lncRNAs of interest. For example, HSALNT0229539 is a 1646 nt-long human lncRNA annotated in the LncBook database (https://ngdc.cncb.ac.cn/lncbook/transcript?transid=HSALNT0229539), which is located at chr16:29679186-29698684 (+) (Figure 4A). The CPAT and CPC2 scores of HSALNT0229539 were 0.286 and 0.209, respectively (Figure 4A). ORFs are covered by more than one line of evidence (Ribo-seq, and TIS) on average (Figure 4B). Only HSALNT0229539 ORF-1 is supported by Pfam evidence (Figure 4B). Detailed sequence information of lncRNA HSALNT0229539 is shown in a popup window after clicking the hyperlink on the “Sequence” arrow (Figure 4C). In total, 5 ORFs were discovered in lncRNA HSALNT0229539, and detailed information is summarized in the following “ORF and peptide information” table (Figure 4B). LncRNA HSALNT0229539 is much more highly expressed in fallopian tube than in other normal human tissues (Figure 4D). Furthermore, HSALNT0229539 is extensively expressed in multiple cancer cell lines, indicating that HSALNT0229539 is a cancer-universally expressed lncRNA (Figure 4E). HSALNT0229539 ORF-1 is located at 19–372 of lncRNA HSALNT0229539, which is predicted to translate as the following peptide: MKQAVRAARQAADFTLKVEVECSSLQEAVQAAEAGADLVLLDNFKPEELHPTATVLKAQFPSVAVEASGGITLDNLPQFCGPHIDVISMGMLTQAAPALDFSLKLFAKEVAPVPKIH (Figure 4F). HSALNT0229539 ORF-1 is predicted with coding potential scores of 0.286 and 0.209 for CPAT and CPC2, respectively. In the Pfam database, QRPTase_C is matched the ORF-1 sequence. In the RPFdb database, 154 Ribo-seq signals were mapped to the ORF-1 region. In addition, two pieces of TIS evidence was found in the HSALNT0229539 ORF-1 region. Evidence from outside public databases can be accessed by clicking the corresponding hyperlinks in the “Database” column.

## Discussion

The rapid development of high-throughput RNA sequencing technologies largely facilitates the discovery and deep investigation of lncRNAs (Atkinson et al., 2012; Brar and Weissman, 2015; Stark and Grzelak, 2019). These RNAs transcribed from typically non-protein-coding regions of genomes have recently been demonstrated to encode functional peptides in various biological contexts. The LncPep database provides an online resource for peptide-encoded lncRNAs and contains 883,804 lncRNAs across 39 species with translational evidence. LncPep offers various ways to browse and search lncRNA-encoding peptide resources and supports users in predicting and blasting customized lncRNA/peptide sequences for exploratory research on novel lncRNA transcripts or peptides. Furthermore, users can download the full datasets deposited in LncPep, which will empower researchers to explore the “coding realm” of lncRNAs. A “document” page is offered for users to understand and use this database quickly.

To date, FuncPEP (Dragomir et al., 2020), ncEP (Liu et al., 2020), cncRNAdb (Huang et al., 2021), and SmProt (Hao et al., 2018) have been developed for noncoding RNA peptides. FuncPEP, ncEP, and cncRNAdb curated experimentally validated peptides encoded by noncoding RNAs, including lncRNAs, circRNAs, and miRNAs. SmProt collected peptides shorter than 100 mino acids (aa) identified from ribosome profiling data, literature, or MS, but no peptides longer than 100 aa encoded by lncRNAs were included (Lun et al., 2020; Meng et al., 2020; Cai et al., 2021). Compared to these existing databases of noncoding RNA-encoded peptides, LncPep is focused on lncRNAs and has four advantages: 1) both validated and predicted lncRNA-encoded peptides are included in LncPep; 2) abundant evidence and detailed annotations are supplied to support peptides and lncRNAs; 3) LncPep does not have a length limitation for peptides, and peptides longer than 100 aa are also important, which has been reported by multiple studies; and 4) the “Predict” and “Blast” modules will help users to explore novel lncRNAs and peptides.

Some limitations still need improvement. Internal ribosome entry sites (IRESs) (Hellen and Sarnow, 2001; Bonnal et al., 2003) are functional cis-acting RNA elements that can direct 40S ribosomes to an internal position on the RNA for translation initiation; thus, IRESs are also an important support for lncRNA translation. RNA structure (Mao et al., 2014; Mauger et al., 2019) also affects its translation; thus, lncRNA structure needs to be taken into account. We did not include these lines of evidence due to the limited available datasets. In the future, we will continue to update the database and add IRES, lncRNA structure, and more Ribo-seq data to support lncRNA translation; we will also further improve the web interface of the database.

## Materials and Methods

### Data Collection

Basic information on lncRNAs was retrieved from LncBook (Ma et al., 2019), NONCODE (Zhao et al., 2021), and LNCipedia (Volders et al., 2019), and 898,452 lncRNA transcripts across 39 species were included. The lncRNA transcript expression profiles in different normal human tissues and multiple types of cancer cell lines were retrieved from LncExpDB (Li et al., 2021) and were previously collected from the Human Protein Atlas (HPA) (Uhlen et al., 2017) and the Cancer Cell Line Encyclopedia (CCLE) (Ghandi et al., 2019), respectively. In particular, the lncRNA expression of normal human tissues included 122 samples in 32 different tissue types, and the expression of cancer cell lines was from 659 cancer cell lines in 44 primary sites.

### Analysis of Evidence for lncRNA-Translated Peptides

The CPAT algorithm and CPC2 were employed to evaluate RNA encoding potential. The CPAT algorithm is based on a logistic regression model built with sequence features from known coding RNA candidates (Wang et al., 2013). We calculated the CPAT scores of lncRNAs for all species, and the CPAT scores were used as one of the criteria to assess the reliability of lncRNA encoding potential. CPC2 is a fast and accurate coding potential calculator based on intrinsic sequence features and is a species-neutral tool (Kang et al., 2017). Thus, the CPC2 scores were calculated for all lncRNA transcripts of 39 species as one line of evidence for encoding potential.

Ribosomes are key modules in polysomes with actively translated RNAs (Ramakrishnan, 2002). Therefore, the association with ribosomes/polysomes detected by ribosome profiling (Ribo-seq) can serve as strong evidence for peptide-translated lncRNAs. RPFdb (Wang et al., 2019) is a public resource for ribosome profiling containing Ribo-seq data from 3,603 samples. We downloaded the Ribo-seq data for humans, mice, *C. elegans*, chicken, rat, zebrafish, and *Arabidopsis thaliana* and then mapped them to ORFs of lncRNA transcripts with coverage >90% by using bedtools. The mapped Ribo-seq signals are evidence of lncRNA translation.

Translation initiation sites (TISs) are important for protein/peptide production from transcripts. Global translation initiation sequencing technology (Wan and Qian, 2014) was used to identify genome-wide TISs. TISdb (Wan and Qian, 2014) is a database that curates human and mouse TISs characterized by global translation initiation sequencing. We downloaded these validated TISs from TISdb and mapped them to ORFs of lncRNA transcripts, and the mapped TISs were used as evidence for lncRNA translation.

The N6-methyladenosine modification of RNA (m6A) is the most abundant internal modification on RNA transcripts in eukaryotic cells. m6A located in 3′ UTRs can promote the translation of capped RNAs (Helm and Motorin, 2017). The RNA EPItranscriptome Collection (REPIC) database (Liu et al., 2019) and m6A-Atlas database (Tang et al., 2021) are two commonly used m6A modification resources. We downloaded and merged the m6A profiles for humans, mice, Arabidopsis, chimpanzees, fruit flies, rats, yeast, and zebrafish from these two databases and mapped them to the 3′ UTRs of lncRNAs. Mapped m6A modification sites are used to support lncRNA translation.

The Pfam database (Mistry et al., 2021) is a large collection of existing protein families and is the most famous database to analyse novel genomes and proteins. Thus, we downloaded the Pfam datasets and applied hmmsearch to search all the predicted lncRNA peptides, and an e-value < 0.0001 was used as the cut-off.

### Peptide Sequence Prediction

The potential peptide sequences translated from candidate lncRNAs were predicted by using Open Reading Frame (ORF) Finder, which searches for ORFs in the DNA sequences of lncRNAs of interest (Wheeler et al., 2003). If peptides overlapped, then we used the longer one. In particular, ORF Finder performs a six-frame translation of DNA sequences of interest and returns candidate ORF sequences. Both ATG and non-ATG parameters were applied in ORF prediction, as non-ATG sequences have been shown to be an important group of translation initiation sites (Ingolia et al., 2011; Lee et al., 2012).

### Calculation of Peptide-Encoding Scores of lncRNA

We defined a peptide-encoding score to quantitatively assess the lncRNA translation potential, which is a summation of the CPAT, CPC2, m6A, Pfam, Ribo-seq, and TIS scores as follows:

$$Score=\sum \left(S_{\left(CPAT\right)},S_{\left(CPC\right)},S_{\left(m6A\right)},S_{\left(Pfam\right)},S_{\left(Ribo-seq\right)},S_{\left(TIS\right)}\right)$$

For m6A, Pfam, Ribo_seq, and TIS, if one sample or sequence mapped to the related peptides, we defined the related score as 1; if no sequence mapped, the score was 0. Scores of these 5 pieces of evidence were calculated as follows:

Score of m6A:

$$S_{\left(m6A\right)}=\frac{Hits\left(m6A\right)}{Median\left(m6A\right)}$$

Score of Pfam:

$$S_{\left(Pfam\right)}=\frac{Hits\left(Pfam\right)}{Median\left(Pfam\right)}$$

Score of Ribo-seq:

$$S_{\left(Ribo-seq\right)}=5\times \frac{Hits\left(Ribo-seq\right)}{Median\left(Ribo-seq\right)}$$

Score of TIS:

$$S_{\left(TIS\right)}=\frac{Hits\left(TIS\right)}{Median\left(TIS\right)}$$

For the CPAT and CPC2, the scores were based on the coding probability that these two algorithms provided. In addition, the scores of CPAT and CPC2 were as follows:

Score of CPAT:

$$S_{\left(CPTA\right)}=CPAT_{\left(coding\_probability\right)}$$

### Score of CPC2

$$S_{\left(CPC2\right)}=CPC2_{\left(coding\_probability\right)}$$

### Database Implementation

LncPep was built with Python FLASK_REST API (https://flask-restful.readthedocs.io/) as the backend web framework. MongoDB (https://www.mongodb.com/) was adopted for data deposition and management in the LncPep database. Angular (https://angular.io/) was utilized to develop web interfaces. Bootstrapping (https://getbootstrap.com/) was employed as the frontend framework, and Echarts (https://echarts.apache.org/) was applied for data visualization. The LncPep database is freely available to all users at http://www.shenglilabs.com/LncPep. The LncPep website is tested and supported in popular web browsers, such as Google Chrome, Microsoft Edge, Firefox, and Safari.

## Data Availability

The original contributions presented in the study are included in the article/Supplementary Material, further inquiries can be directed to the corresponding author.
